# The optimal pulse pressures for healthy adults with different ages and sexes correlate with cardiovascular health metrics

**DOI:** 10.3389/fcvm.2022.930443

**Published:** 2022-12-05

**Authors:** Chung-Hsing Chou, Jiu-Haw Yin, Yu-Kai Lin, Fu-Chi Yang, Ta-Wei Chu, Yuan Chieh Chuang, Chia Wen Lin, Giia-Sheun Peng, Yueh-Feng Sung

**Affiliations:** ^1^Department of Neurology, Tri-Service General Hospital, National Defense Medical Center, Taipei, Taiwan; ^2^Graduate Institute of Medical Sciences, National Defense Medical Center, Taipei, Taiwan; ^3^Division of Neurology, Department of Internal Medicine, Taipei Veterans General Hospital, Hsinchu, Taiwan; ^4^Department of Obstetrics and Gynecology, Tri-Service General Hospital, National Defense Medical Center, Taipei, Taiwan; ^5^MJ Health Screening Center, Taipei, Taiwan; ^6^MJ Health Research Foundation, MJ Group, Taipei, Taiwan

**Keywords:** pulse pressure, arterial stiffness, cardiovascular disease, health metrics, health score

## Abstract

**Background:**

Pulse pressure (PP) may play a role in the development of cardiovascular disease, and the optimal PP for different ages and sexes is unknown. In a prospective cohort, we studied subjects with favorable cardiovascular health (CVH), proposed the mean PP as the optimal PP values, and demonstrated its relationship with healthy lifestyles.

**Methods and results:**

Between 1996 and 2016, a total of 162,636 participants (aged 20 years or above; mean age 34.9 years; 26.4% male subjects; meeting criteria for favorable health) were recruited for a medical examination program. PP in male subjects was 45.6 ± 9.4 mmHg and increased after the age of 50 years. PP in female subjects was 41.8 ± 9.5 mmHg and increased after the age of 40 years, exceeding that of male subjects after the age of 50 years. Except for female subjects with a PP of 40–70 mmHg, PP increase correlates with both systolic blood pressure (BP) increase and diastolic BP decrease. Individuals with mean PP values are more likely to meet health metrics, including body mass index (BMI) <25 kg/m^2^ (chi-squared = 9.35, p<0.01 in male subjects; chi-squared = 208.79, *p* < 0.001 in female subjects) and BP <120/80 mmHg (chi-squared =1,300, *p* < 0.001 in male subjects; chi-squared =11,000, *p* < 0.001 in female subjects). We propose a health score (Hscore) based on the sum of five metrics (BP, BMI, being physically active, non-smoking, and healthy diet), which significantly correlates with the optimal PP.

**Conclusion:**

The mean PP (within ±1 standard deviation) could be proposed as the optimal PP in the adult population with favorable CVH. The relationship between health metrics and the optimal PP based on age and sex was further demonstrated to validate the Hscore.

## Introduction

Pulse pressure (PP) is defined as the difference between systolic and diastolic blood pressures (BPs), and it correlates with the elastic properties of the arterial wall and cardiac volume. A higher PP frequently reflects increased arterial stiffness due to atherosclerosis or general aging (1). PP is recognized as a potential risk factor for cardiovascular diseases (CVDs), such as myocardial infarction, stroke, cardiovascular mortality (2–4), and cognitive decline (5). The importance of PP in determining cardiovascular risk is based on the fact that PP is a marker of large artery stiffness, an independent predictor of cardiovascular events (6). PP has important predictive values for CVD among people aged ≥60 years but only a marginal predictive value for people aged <60 years (7). The clinical significance of PP in young people is still controversial, although a high PP might carry a reduced risk of cerebrovascular events in young and middle-aged subjects with hypertension (8). Moreover, a study with long-term follow-up revealed that younger and middle-aged adults with idiopathic spontaneous hypertension had a higher relative risk for CVD and congestive heart disease than those with optimal-normal BP (9). For specific patient groups, such as those with both type 2 diabetes and CVD compared to those without CVD, PP has been shown to be more relevant than systolic and diastolic BP (10). PP measurement is convenient in routine medical settings, and it might not only provide a quick means of estimating CVD risk but also may have significant clinical value for predicting CVD outcomes. Compared to the arterial stiffness index measured by finger photoplethysmography, PP appears to have greater clinical value for predicting CVD and mortality outcomes (6). Validation of the optimal PP for individuals of different ages and sexes is essential for applying PP to health assessments in a broader population. Participants with favorable cardiovascular health (CVH) from a community-based database may have relatively normal BP values and could be recruited for an investigation of optimal PP and health metrics. It has been shown that meeting a greater number of CVH metrics recommended by the American Heart Association (e.g., not smoking; being physically active; having normal BP, blood glucose and total cholesterol levels, and weight; and eating a healthy diet) is associated with a lower risk of total and CVD mortality (11).

The present study proposes the mean PP of participants with favorable CVH as the optimal PP. The relationship between PP and biomarkers, such as lipid profiles, is also studied. Results of the optimal PP values are validated by investigating whether individuals with optimal PP meet more health metrics and we further demonstrate that those meeting more health metrics are more likely to have the optimal PP.

## Methods

### Participant selection

This study was based on an ongoing large prospective cohort whose details were described elsewhere (12, 13). In brief, this cohort study recruited around 1.3 million participants between 1996 and 2016. A private health screening firm, the MJ Health Management Institution, provided a standard medical screening program (available at www.mjhrf.org/file/en/report/). Participants were Taiwanese of Chinese descent. MJ members are subjected to periodic and comprehensive physical assessments, which include anthropometric measurements, spirometry tests, blood and urinary tests, and imaging studies, as well as a standard self-administered lifestyle questionnaire survey, which includes smoking status [current smoking (0 points) vs. never and former (1 point)], physical activity [inactive (0 points) vs. active (1 point)], healthy diet score [<2 components (0 points) vs. ≥2 components (1 point)] and menopause age if post-menopausal. Consent was secured from all participants, and individuals with hypertension, defined as systolic blood pressure (SBP) ≥140 mmHg, diastolic blood pressure (DBP) ≥90 mmHg, treated or self-reported hypertension, or dyslipidemia, defined as total cholesterol (CHOL) ≥200 mg/dL, triglyceride (TG) ≥150 mg/dL, low-density lipoprotein cholesterol (LDL-C) ≥130 mg/dL or high-density lipoprotein cholesterol (HDL-C) <40 mg/dL, treated or self-reported dyslipidemia were excluded. Those with a history of diabetes, thyroid disorder, asthma, interstitial pulmonary disease, chronic obstructive pulmonary disease, cystic fibrosis, nephritis, hepatitis, cirrhosis, stroke, or abnormality on EKG were also excluded. The study protocols (2021-04-015BC) were approved by the Institutional Review Board of the Taipei Veterans General Hospital (Taipei, Taiwan).

### Definitions for the study protocol

We use “How often did you exercise during the last 2 weeks?” to define physical activity as “inactive” for none or rarely and as “active” for others. The healthy diet score is calculated by summing the following components, assigning each for the consumption of fruits and vegetables (≥4.5 cups/day), fish (≥two 3.5-oz servings/wk), fiber-rich whole grains (≥three 1-oz–equivalent servings/day), sodium (<1,500 mg/day), and sugar-sweetened beverages (<36 oz/wk). The body mass index (BMI) is calculated as weight in kilograms divided by height in meters squared [≥25 (0 points) vs. <25 (1 point) kg/m^2^].

### BP measurements and PP calculation

After at least 5 min of rest, systolic and diastolic BPs were measured from the non-dominant arm at the MJ assessment center using an automated BP device (Omron HEM-7201 or GE Dinamap ProCare 100) or manually using a sphygmomanometer with an inflatable cuff in combination with a stethoscope if the BP device failed to measure the BP. All measurements were performed while the participant was seated and were carried out by nurses who had received BP measurement training. PP was calculated by subtracting the diastolic from the systolic BP value.

### Statistical analysis

Continuous variables were expressed as mean ± standard deviation (SD). Variables were compared between male and female groups using Student's *t*-test. The *F*-test was conducted to examine the variance between PP (mean ± 1 SD or 2 SD) and age in both male and female groups. The correlation coefficient *r* measurements were conducted between stratified PP and clinical and laboratory data, including SBP and DBP, for male and female groups. The chi-square test of independence was conducted to identify statistically significant relationships between PP and the five metrics, including smoking, physical activity, healthy diet score, BMI, and BP. We generated a health score (Hscore), adapted from established CVH profiles, based on the sum of the points from BMI and BP measurements [SBP ≥120 mmHg or DBP ≥80 mmHg (0 points) vs. others (1 point)] or all the five-health metrics (BMI, BP, smoking, physical activity, and healthy diet score) and conducted the *F*-test of equality of variances. STATA/SE 14.2 was used for the statistical analysis.

## Results

A total of 1,398,265 participants (49.4% male subjects) aged 20 years or above were recruited during 1996–2016. We excluded 372,879 participants with incomplete information. Among the remaining 1,025,386 participants (44.9% male subjects), 162,636 (26.4% male subjects) individuals fulfilled the inclusion criteria of favorable health including the CVH indexes and were included in the present analysis.

The mean ages of male and female subjects were 34.5 ± 9.3 and 35.0 ± 8.3 years old, respectively. Most participants (40.59% male subjects; 44.71% female subjects) were aged between 30 and 39 years at the time of their examinations. The mean BMI of male and female subjects were 22.4 ± 2.7 kg/m^2^ and 21.0 ± 2.7 kg/m^2^, respectively. The clinical and laboratory characteristics of participants are shown in Table 1.

**Table 1:** Clinical and laboratory characteristics of participants.

	**Male**		**Female**		**Total**		***T*-test**
	**Mean**	**SD**	**Mean**	**SD**	**Mean**	**SD**	
Age, year	34.5	(9.3)	35.0	(8.3)	34.9	(8.6)	−11.22^***^
BMI, kg/m^2^	22.4	(2.7)	21.0	(2.7)	21.3	(2.8)	92.74^***^
WC, cm	77.2	(7.3)	68.3	(6.2)	70.7	(7.6)	242.31^***^
SBP, mmHg	115.0	(11.2)	106.5	(11.5)	108.7	(12.0)	131.98^***^
DBP, mmHg	69.4	(8.2)	64.7	(8.4)	65.9	(8.6)	100.69^***^
PP, mmHg	45.6	(9.4)	41.8	(9.5)	42.8	(9.6)	70.75^***^
FPG, mg/dL	96.0	(7.1)	92.2	(7.0)	93.2	(7.2)	93.80^***^
AST, IU/L	19.4	(3.3)	17.7	(3.3)	18.1	(3.4)	93.52^***^
ALT, IU/L	19.6	(6.1)	14.6	(5.1)	15.9	(5.8)	164.70^***^
GGT, IU/L	18.0	(7.6)	12.5	(5.4)	13.9	(6.5)	161.62^***^
eGFR, mL/min/1.73 m^2^	86.3	(12.8)	90.1	(60.3)	89.1	(52.2)	−12.71^***^
TG, mg/dL	79.9	(27.6)	67.7	(24.6)	70.9	(26.0)	84.95^***^
CHOL, mg/dL	169.2	(19.1)	169.2	(19.1)	169.2	(19.1)	0.61
HDL-C, mg/dL	54.7	(10.1)	61.8	(12.5)	59.9	(12.3)	−110.00^***^
LDL-C, mg/dL	98.7	(18.2)	93.8	(18.3)	95.1	(18.4)	47.89^***^
TSH, μIU/mL	1.44	(0.73)	1.54	(0.81)	1.52	(0.79)	−23.53^***^
Number	42,944 (26.4%)		119,692 (73.6%)		162,636 (100.0%)		

Data are given as the number (percent) for sexes; for all the other variables, mean ± standard deviation (SD) is reported. BMI indicates body mass index; WC, waist circumference; SBP, systolic blood pressure; DBP, diastolic blood pressure; PP, pulse pressure; FPG, fasting plasma glucose; AST, aspartate aminotransferase; ALT, alanine aminotransferase; GGT, gamma-glutamyl transferase; eGFR, estimated glomerular filtration rate; TG, triglyceride; CHOL, total cholesteral; HDL-C, high-density lipoprotein cholesterol; LDL-C, low-density lipoprotein cholesterol; TSH, thyroid-stimulating hormone.

*** p < 0.001.

### PP in female subjects exceeds that of male subjects after the age of 50

**Figure 1:**
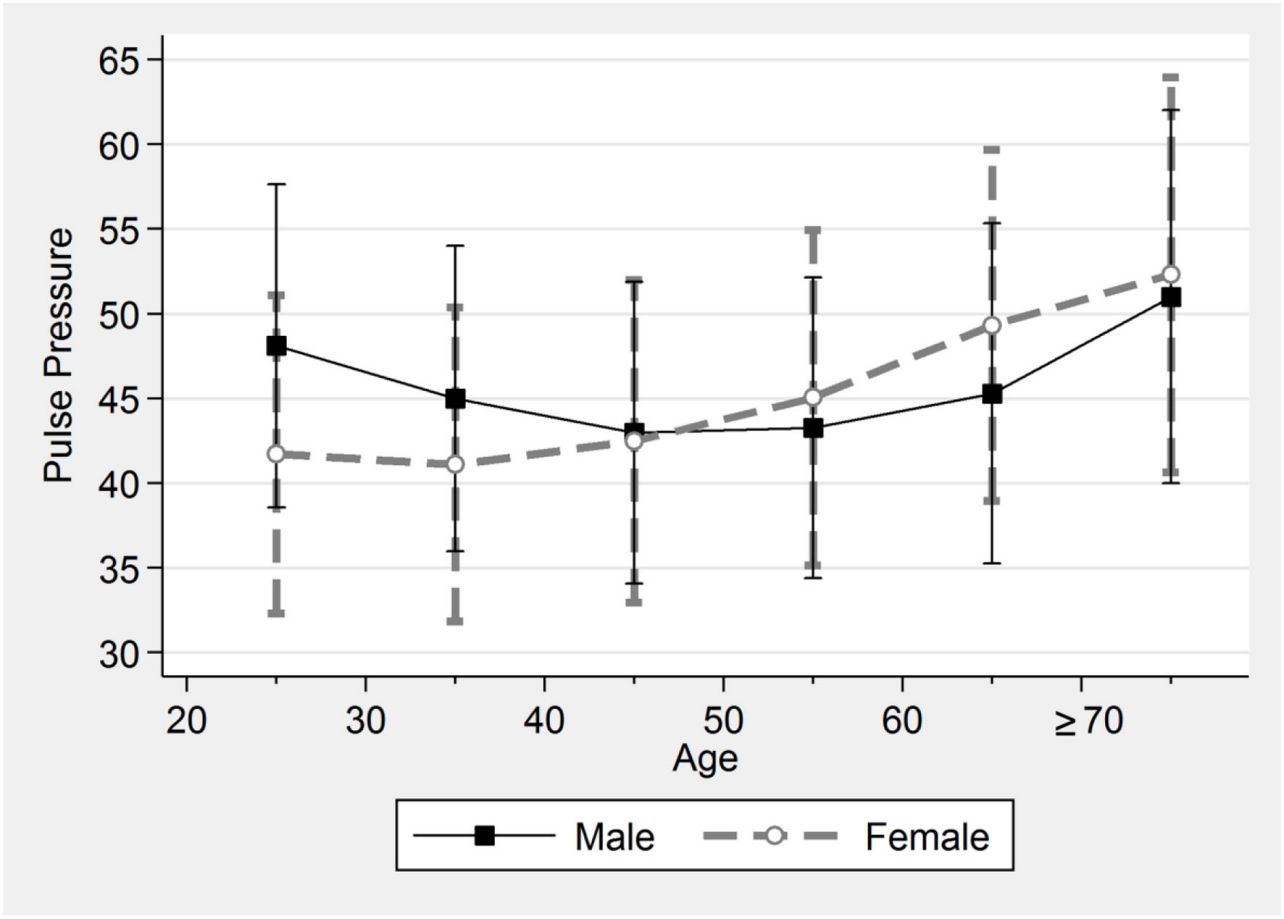
Pulse pressure by sex and age. The J-shape curves demonstrate the non-linear relationship between age and PP for male and female subjects. PP in female subjects becomes greater than in male subjects after the age of 50 years.

The mean PP of all participants was 42.8 ± 9.6 mmHg and increased after the age of 40 years (Table 2). PP (mean ± 1 SD) in male subjects was 45.6 ± 9.4 mmHg and increased after the age of 50 years. PP in female subjects was 41.8 ± 9.5 mmHg and increased after the age of 40 years. Notably, the mean PP in female subjects (45.0 ± 9.9 mmHg) exceeded that of male subjects (43.3 ± 8.9 mmHg) after the age of 50 years. The J-shape curves in Figure 1 demonstrate the non-linear relationship between age and PP and differences between sexes. The calculated intersection point of the two curves for female and male subjects individually was 47.2 years old, at PP 43.0 mmHg. This was close to the average menopause age of 48.4 ± 4.3 years, based on questionnaire results from those who had undergone menopause, representing 4.43% of all female subjects.

**Table 2:** Distribution of pulse pressure across all age groups and sexes.

**Age**	**Male**						**Female**						**Total**					
	***N***	**%**	**Mean**	**SD**	±**1 SD**	±**2 SD**	***N***	**%**	**Mean**	**SD**	±**1 SD**	±**2 SD**	***N***	**%**	**Mean**	**SD**	±**1 SD**	±**2 SD**
≤ 29	14,086	32.80%	48.3	(9.6)	38.7~57.9	29.1~67.5	32,684	27.31%	41.8	(9.4)	32.4~51.2	23.0~60.6	46,770	28.76%	43.8	(9.9)	33.9~53.7	24.0~63.6
30–39	17,432	40.59%	45.0	(9.0)	36.0~54.0	27.0~63.0	53,509	44.71%	41.1	(9.3)	31.8~50.4	22.5~59.7	70,941	43.62%	42.1	(9.4)	32.7~51.5	23.3~60.9
40–49	8,475	19.74%	43.0	(8.9)	34.1~51.9	25.2~60.8	27,554	23.02%	42.5	(9.5)	33.0~52.0	23.5~61.5	36,029	22.15%	42.6	(9.4)	33.2~52.0	23.8~61.4
50–59	2,431	5.66%	43.3	(8.9)	34.4~52.2	25.5~61.1	5,323	4.45%	45.0	(9.9)	35.1~54.9	25.2~64.8	7,754	4.77%	44.5	(9.6)	34.9~54.1	25.3~63.7
60–69	470	1.09%	45.3	(10.0)	35.3~55.3	25.1~65.3	593	0.50%	49.3	(10.4)	38.9~59.7	28.5~70.1	1,063	0.65%	47.5	(10.4)	37.1~57.9	26.7~68.3
≥70	50	0.12%	51.0	(11.0)	40.0~62.0	29.0~73.0	29	0.02%	52.3	(11.6)	40.7~63.9	29.1~75.5	79	0.05%	51.5	(11.2)	40.3~62.7	29.1~73.9
Total	42,944	100.00%	45.6	(9.4)	36.2~55.0	26.8~64.4	119,692	100.00%	41.8	(9.5)	32.3~51.3	22.8~60.8	162,636	100.00%	42.8	(9.6)	33.2~52.4	23.6~62.0
*F*-test	434.01^***^						297.77^***^						298.91^***^					

Data of pulse pressure (mmHg) are given as mean ± standard deviation (SD).

*** p < 0.001.

**Figure 2:**
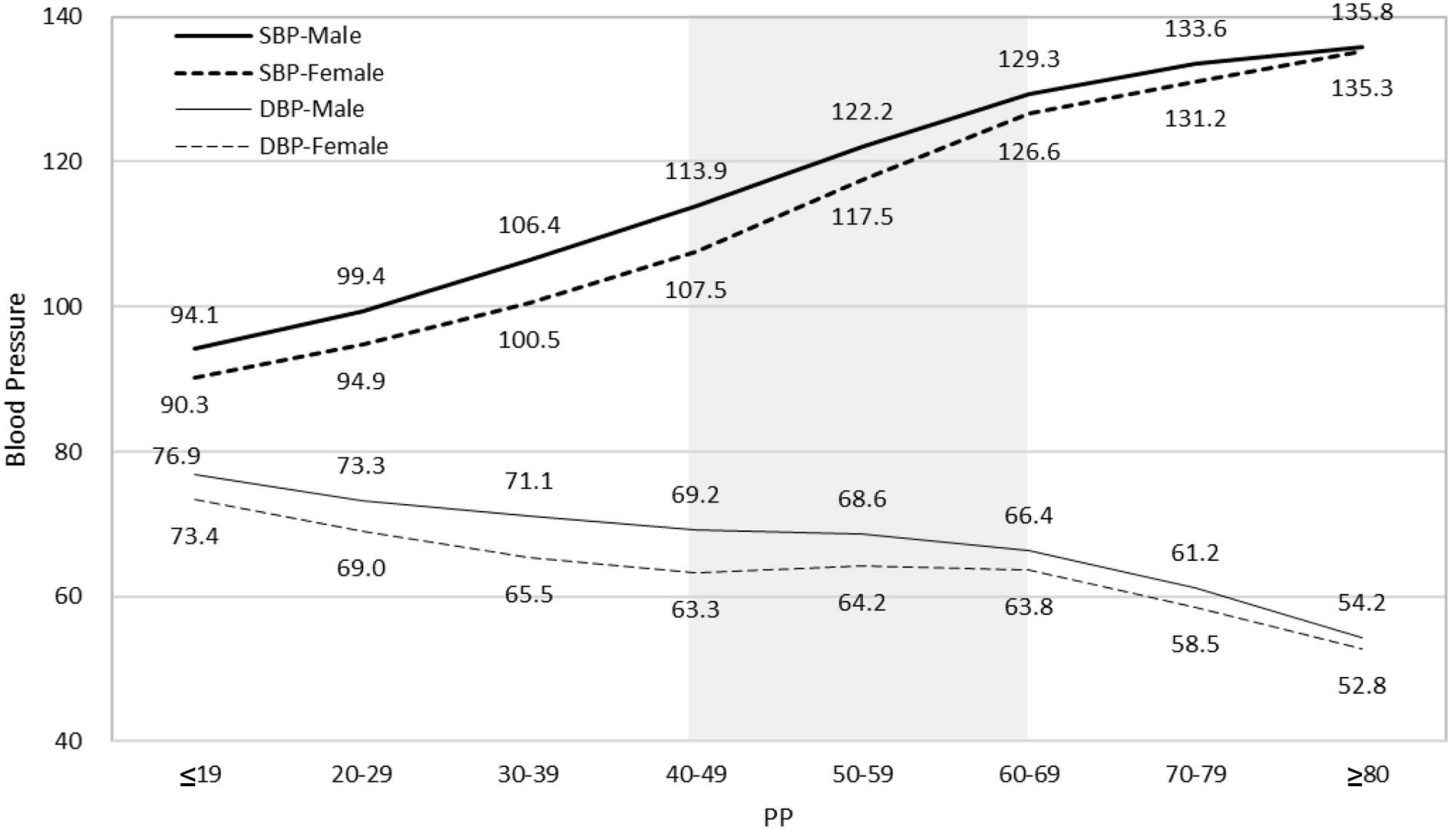
Relationship of PP with SBP and DBP for both sexes. PP increase always results from both SBP increase and DBP decrease except in female subjects with PP 40–70 mmHg (shadow area). Mean DBP of female subjects does not decrease as PP increases from 40 mmHg to 70 mmHg. PP indicates pulse pressure; SBP, systolic blood pressure; DBP, diastolic blood pressure.

The mean SBP of male and female subjects was 115.0 ± 11.2 mmHg and 106.5 ± 11.5 mmHg, respectively. The mean DBP of male and female subjects was 69.4 ± 8.2 mmHg and 64.7 ± 8.4 mmHg (Table 1 and Supplementary Figure S1), respectively. In general, the increase in PP results from both SBP increase and DBP decrease. However, DBP did not decrease in female subjects with PP from 40 to 70 mmHg (Figure 2). As a result, we first demonstrate a specific range of PP, 40 to 70 mmHg in female subjects, in which the PP increase is only due to an increase in SBP rather than a decrease in DBP.

### Association between PP and the clinical and laboratory characteristics

Systolic blood pressure is significantly correlated with PP for both male (*r* = 0.6916, *p* < 0.05) and female (*r* = 0.6885, *p* < 0.05) subjects (Supplementary Table S1). DBP in female subjects does not decrease as PP increases from 40 to 70 mmHg although there is a negative correlation between DBP and PP (*r* = −0.1696, *p* < 0.05). In contrast, DBP in male subjects decreases continuously as PP increases, and it correlates relatively well with PP (*r* = −0.1925, *p* < 0.05). There is a positive correlation between BMI and PP, and the correlation coefficient for female subjects (*r* = 0.1261, *p* < 0.05) is greater than for male subjects (*r* = 0.0728, *p* < 0.05). Although waist circumference (WC) in female subjects (68.3 ± 6.2 cm) is less than in male subjects (77.2 ± 7.3 cm), the positive correlation between WC and PP in female subjects (*r* = 0.1133, *p* < 0.05) is greater than in male subjects (*r* = 0.0325, *p* < 0.05). A positive correlation between PP and the lipid profile, including TG, CHOL, and LDL-C, can be found in female subjects, but there is a negative correlation between PP and the lipid profile in male subjects.

### Association between PP and health metrics

Pulse pressure significantly correlates with health metrics, including BMI <25 kg/m^2^ (chi-squared = 9.35, *p* < 0.01 in male subjects; chi-squared = 208.79, *p* < 0.001 in female subjects;) and BP <120/80 mmHg (chi-squared = 1,300, *p* < 0.001 in male subjects; chi-squared = 11,000, *p* < 0.001 in female subjects) (Table 3). A higher proportion of BP <120/80 mmHg could be found in male subjects (68.01%) and female subjects (90.71%) with the optimal PP between ±1 SD than in groups with suboptimal PP between ±1~2 SD (51.10% of male subjects and 74.20% of female subjects) or out of ±2 SD (43.36% of male subjects and 45.03% of female subjects (Table 3A). Conversely, the proportion of BP ≥120/80 mmHg was higher in male subjects (56.64%) and female subjects (54.97%) with PP out of ±2 SD than the groups with PP between ±1~2 SD and the groups with the optimal PP between ±1 SD. A higher proportion of BMI <25 kg/m^2^ could be found in male subjects (84.65%) and female subjects (93.12%) with the optimal PP between ±1 SD, and they received 1 point based on BMI measurement. Conversely, the proportion of BMI ≥25 kg/m^2^ was 15.11% in male subjects and 9.80% in female subjects with PP out of ±2 SD, and they got 0 points (Table 3A).

**Table 3:** Pulse pressure (PP) significantly correlates with health metrics, BMI <25 kg/m^2^ and BP <120/80 mmHg.

	**Male**				**Female**			
	**PP between** ±**1 SD**	**PP between** ±**1**~**2 SD**	**PP out of** ±**2 SD**	**Total**	**PP between** ±**1 SD**	**PP between** ±**1**~**2 SD**	**PP out of** ±**2 SD**	**Total**
**(A)**								
Blood pressure	χ^**2**^ = 1,300^***^				χ^**2**^ = 11,000^***^			
BP ≥ 120/80	9,462	5,570	1,121	16,153	7,382	8,964	3,006	19,352
	31.99%	48.90%	56.64%	37.61%	9.29%	25.80%	54.97%	16.17%
BP < 120/80	20,112	5,821	858	26,791	72,095	25,783	2,462	100,340
	68.01%	51.10%	43.36%	62.39%	90.71%	74.20%	45.03%	83.83%
BMI	χ^**2**^ = 9.35^**^				χ^**2**^ = 208.79^***^			
BMI ≥ 25	4,541	1,885	299	6,725	5,470	3,171	536	9,177
	15.35%	16.55%	15.11%	15.66%	6.88%	9.13%	9.80%	7.67%
BMI < 25	25,033	9,506	1,680	36,219	74,007	31,576	4,932	110,515
	84.65%	83.45%	84.89%	84.34%	93.12%	90.87%	90.20%	92.33%
Total	29,574	11,391	1,979	42,944	79,477	34,747	5,468	119,692
	100.00%	100.00%	100.00%	100.00%	100.00%	100.00%	100.00%	100.00%
**(B)**								
Blood pressure	χ^**2**^ = 1,300^***^				χ^**2**^ = 11,000^***^			
BP ≥ 120/80	9,462	5,570	1,121	16,153	7,382	8,964	3,006	19,352
	58.58%	34.48%	6.94%	100%	38.15%	46.32%	15.53%	100%
BP < 120/80	20,112	5,821	858	26,791	72,095	25,783	2,462	100,340
	75.07%	21.73%	3.20%	100%	71.85%	25.70%	2.45%	100%
BMI	χ^**2**^ = 9.35^**^				χ^**2**^ **=** 208.79^***^			
BMI ≥ 25	4,541	1,885	299	6,725	5,470	3,171	536	9,177
	67.52%	28.03%	4.45%	100%	59.61%	34.55%	5.84%	100%
BMI < 25	25,033	9,506	1,680	36,219	74,007	31,576	4,932	110,515
	69.12%	26.25%	4.64%	100%	66.97%	28.57%	4.46%	100%
Total	29,574	11,391	1,979	42,944	79,477	34,747	5,468	119,692

BMI indicates body mass index; SD, standard deviation.

** p < 0.01;

*** p < 0.001.

The proportion of the optimal PP between ±1 SD in male subjects (75.07%) and female subjects (71.85%) who received 1 point based on BP <120/80 mmHg was higher than that among those who received 0 points (58.58% of male subjects and 38.15% of female subjects) (Table 3B). Conversely, the proportion of PP out of ±2 SD among male subjects (6.94%) and female subjects (15.53%) who received 0 points based on BP ≥120/80 mmHg was higher than that among those with BP <120/80 mmHg who got 1 point. The proportion of the optimal PP between ±1 SD in male subjects (69.12%) and female subjects (66.97%) who received 1 point based on BMI <25 kg/m^2^ was higher than that among those who received 0 points (67.52% of male subjects and 59.61% of female subjects). Conversely, among those with PP out of ±2 SD, the proportion of BMI ≥25 kg/m^2^ was 4.45% in male subjects and 5.84% in female subjects (Table 3B). Individuals with the optimal PP, i.e., PP between ±1 SD, are therefore presumed to meet healthy metrics, such as BMI and BP, and individuals meeting the healthy metrics are more likely to have the optimal PP.

Subjective self-reporting on the lifestyle questionnaire (including non-smoking, physically active, and having a healthy diet) found no significant difference between male subjects with PP between ±1 SD, PP between ±1~2 SD, and out of ±2 SD (Supplementary Table S2A). Furthermore, a lower proportion of those who received 1 point based on meeting the health metrics was found in female subjects with the optimal PP between ±1 SD. In female subjects, smoking (chi-squared = 48.76, *p* < 0.001), physical activity (chi-squared = 101.99, *p* < 0.001), and healthy diet (chi-squared = 121.80, *p* < 0.001) were significantly correlated with PP (Supplementary Table S2A). Similarly, the proportion of participants with the optimal PP between ±1 SD was not significantly higher in those who met subjectively measured healthy metrics. Instead, a lower proportion of PP between ±1 SD was found in female subjects who met healthy metrics and received 1 point, including non-smoking (66.28%), physically active (65.33%), or having a healthy diet (65.57%), compared to those who received 0 points (Supplementary Table S2B). Multiple stepwise regression analysis for several variables of PP was further conducted to show that sex, age, and BMI were significantly associated with PP in different models (Supplementary Table S3).

### The generated health score (Hscore) correlates with the optimal PP

In Table 4, combining cutoff scores, including 1 point for BP <120/80 mmHg and 1 point for BMI <25 kg/m^2^, the sum of points among male subjects with the optimal PP between ±1 SD (1.5 ± 0.6) are significantly higher than those with PP between ±1~2SD (1.3 ± 0.7) or out of ±2SD (1.3 ± 0.6) (*F* = 430.00, *p* < 0.001). Similarly, the point scores among female subjects with the optimal PP between ±1 SD (1.8 ± 0.4) are significantly higher than those with PP between ±1~2SD (1.7 ± 0.6) or out of ±2SD (1.4 ± 0.6) (*F* = 4221.20, *p* < 0.001).

**Table 4:** Health score (Hscore) significantly correlates with pulse pressure (PP).

	**PP between** ±**1 SD**		**PP between** ±**1**~**2 SD**		**PP out of** ±**2 SD**		***F*-test**
	**Mean**	**SD**	**Mean**	**SD**	**Mean**	**SD**	
**Male**							
Hscore (0–2)	1.5	(0.6)	1.3	(0.7)	1.3	(0.6)	430.00^***^
Hscore 24 (0–5)	3.2	(1.1)	3.0	(1.2)	2.9	(1.2)	132.66^***^
Hscore 25 (0–5)	3.3	(1.1)	3.1	(1.1)	3.0	(1.1)	147.70^***^
Hscore 27 (0–5)	3.4	(1.1)	3.2	(1.1)	3.1	(1.1)	152.15^***^
Hscore 30 (0–5)	3.4	(1.0)	3.3	(1.1)	3.2	(1.1)	155.64^***^
Number	29,574		11,391		1,979		
**Female**							
Hscore (0–2)	1.8	(0.4)	1.7	(0.6)	1.4	(0.6)	4221.20^***^
Hscore 24 (0–5)	3.7	(0.9)	3.5	(1.0)	3.2	(1.1)	811.34^***^
Hscore 25 (0–5)	3.7	(0.9)	3.6	(1.0)	3.2	(1.1)	794.93^***^
Hscore 27 (0–5)	3.8	(0.9)	3.6	(1.0)	3.3	(1.0)	766.58^***^
Hscore 30 (0–5)	3.8	(0.9)	3.7	(1.0)	3.3	(1.0)	750.34^***^
Number	79,477		34,747		5,468		

Several cutoff scores for body mass index, including 24, 25, 27, and 30 kg/m^2^, were examined. SD indicates standard deviation.

*** p < 0.001.

When all five metrics are combined (including 1 point for BP, 1 point for BMI, 1 point for not currently smoking, 1 point for being physically active, and 1 point for having at least two healthy diet components), the points among those with the optimal PP between ±1 SD are significantly higher (meeting more health metrics) than those with PP between ±1~2SD or out of ±2SD, in both male and female groups (Table 4). The metric scores (the Hscore) using several cutoff scores for BMI, including 24, 25, 27, and 30 kg/m^2^, were all shown to be significantly correlated with PP (*p* < 0.001).

## Discussion

### Principal findings

We studied associations of PP with the clinical and laboratory characteristics in 162,636 participants who met the inclusion criteria of favorable health, free of dyslipidemia, diabetes, and hypertension. We first proposed the mean PP of these participants with favorable CVH as the optimal PP for different ages and sexes. There is an intersection point at 47.2 years of the two curves of mean PP across all ages for male and female subjects (Figure 1). Notably, after the age of 50, PP in female subjects (45.0 ± 9.9 mmHg) grew more than in male subjects (43.3 ± 8.9 mmHg). Second, we observed that PP increase may not always result from both SBP increase and DBP decrease. SBP increase alone contributes to PP increase in female subjects with PP 40–70 mmHg (Figure 2). Third, a higher proportion of individuals who had the optimal PP, i.e., the mean PP ± 1 SD, met more healthy metrics, including BP <120/80 mmHg and BMI <25 kg/m^2^. Lastly, people meeting more health metrics, with a higher Hscore based on both objectively measured values and subjective questionnaire results, were more likely to have the optimal PP.

### The optimal PP may vary between ages and sexes

This study shows that PP among individuals with favorable CVH decreases before the age of 50 years in male subjects and 40 years in female subjects, at which time PP increases. The optimal PP and its predictive value in estimating the risk of CVD may accordingly be associated with age and sex. Age and sex differences in cardiac characteristics including cardiac output have been demonstrated among master athletes (14), and higher cardiac output in male subjects than female subjects may be related to higher PP in our young male subjects. Indeed, age and sex are significantly associated with PP in our multiple stepwise regression models (Supplementary Table S3).

Supplementary Figure S1 shows that high SBP in young male subjects accounts for greater PP compared to female subjects. DBP increases until 50 years of age and thereafter declines in our male group, which is consistent with a study based on the IDACO database (15), but DBP in our older female subjects does not decrease. This may partially explain why high PP and low mean BP have been regarded as favorable features in young adults (9, 16, 17). Indeed, PP may be a better index of arterial stiffness or atherosclerosis than simple SBP or DBP values, and it could be split into an “elastic” component (elPP) and a “stiffening” component (stPP). Elastin and collagen are the major constituents of the extracellular matrix in the media of the central elastic arteries, and their differential properties may be the fundamental determinants of predictive values of PP for risks of CVD (15). Recently, elPP but not stPP was shown to be predictive of total and CVD mortality in a rural Japanese population (18). In terms of affecting the PP of individuals of different ages and sexes, it remains to be determined how components of elPP and stPP change with aging.

Our results are in line with the Hypertension Ambulatory Recording Venetia Study (HARVEST) that, among subjects aged <45 years, PP in male subjects does not increase until 42–45 years, as opposed to around 40 years in female subjects (8). PP of the young-to-middle-aged subjects in our cohort with favorable CVH is lower than the general population screened for stage 1 hypertension in HARVEST, in which high PP carries a reduced risk of hypertension (8). Further studies are required to confirm whether high PP is associated with a lower risk of CVD before PP begins to increase at the ages of 50 and 40, respectively, in male and female subjects.

The mean PP ± 1 SD (42.8 ± 9.6 mmHg) of all participants in our cohort is lower than that of the UK Biobank, a presumably healthier community-based population (50.98 ± 13.2 mmHg; mean age 56.8 years; 45.8% male subjects) in which CVD risk increases 3.8% per 10 mmHg PP increase (6). In addition, hypertensive patients had a 17% increased risk of CVD per 10 mmHg PP increase in a study of older individuals (average 67–72 years), where PP rather than mean BP was found to better determine the risk of CVD (19). Higher CVH score with more favorable CVH metrics was found to be associated with lower baseline and follow-up brachial-ankle pulse wave velocity (baPWV), and it was shown to predict the annual change in baPWV in men and individuals older than 50 years (20). More studies are required to demonstrate if the predictive value of PP for CVD risk is higher for male subjects older than 50 years and female subjects aged 40 years or above in our study cohort. A positive correlation between PP and CVD risk may, therefore, be more evident in a population older than the ages at which PP begins to increase.

Our data provide new insight into the influence of DBP on PP, especially for female subjects whose DBP did not decrease as PP increased from 40 to 70 mmHg (Figure 2 and Supplementary Table S1). Different PP values across all ages for both sexes with favorable CVH may have to be taken into consideration when establishing DBP treatment targets. Low DBP did not show significant effects on cardiovascular risk or primary prevention of stroke in the high-risk Systolic Blood Pressure Intervention Trial (SPRINT) population (21, 22). Conversely, several earlier studies suggested that lower DBP or intensive DBP reduction may increase the risk of coronary artery disease (23, 24). A recent study further suggests that low DBP (<60 mmHg) was associated with an increased risk of composite events among patients aged 20 years or older, with ischemic stroke or transient ischemic attack (25). In a population of veterans aged 45 or older, reduction of DBP below 70 mmHg was associated with increased all-cause mortality (26). A minimum BP target should, therefore, be included in hypertension guidelines. Recently, the Reasons for Geographic and Racial Disparities in Stroke (REGARDS) study found that better CVH in participants free of baseline hypertension was associated with a lower risk of incident hypertension using a 130/80 mm Hg hypertension threshold (27). As DBP goals should be tailored to a patient's individual characteristics, our cohort for studying the optimal PP provides reference DBP values for different ages and sexes.

This study observed differential relationships between PP and serum cholesterol levels in female and male subjects. Patients with hypercholesterolemia have a higher central PP and stiffer blood vessels (28). Our male cohort with favorable CVH nevertheless shows a slightly negative correlation between PP and TG, LDL-C, and CHOL levels although there is a positive correlation between PP and the lipid profile in female subjects. Furthermore, our data correspond with the hypertension cohort of the China Stroke Primary Prevention Trial (CSPPT), in which HDL-C was inversely associated with arterial stiffness, measured by baPWV (29). Similar to our results, TG levels have been shown to be correlated with arterial stiffness, measured as the cardio-ankle vascular index, in a Czech general population aged between 25 and 64 years in the Kardiovize Brno 2030 study (30). Furthermore, among a relatively healthy Taiwanese population, age and hypertension, rather than other metabolic risk factors, were independently associated with silent brain infarctions (31). A cross-sectional study for healthy Korean women aged 44–56 years suggests that changes in BP during the menopausal transition are significant, which may be associated with lipid metabolism and, accordingly, arterial stiffness (32). In our study cohort, the PP of female subjects increased after the age of 40 years and exceeded that of male subjects after the age of 50 years, which is close to the mean menopause age (48.4 ± 4.3 years). According to a cross-sectional analysis based on the Canadian Longitudinal Study on Aging (CLSA), menopause is associated with an increased risk of metabolic syndrome, independent of age (33). Elevated BP is one of the criteria for metabolic syndrome. Interestingly, the SBP of study individuals with or without menopause was 121.9 ± 17.5 mmHg and 113.5 ± 14.9 mmHg, respectively, with a statistically significant difference. In contrast, the DBP of the two groups was 72.2 ± 9.5 mmHg and 72.7 ± 9.5 mmHg, respectively, without statistically significant difference (33). Further studies are required to investigate the role of menopause in our findings that DBP does not decrease in female subjects with PP from 40 to 70 mmHg.

### People with the optimal PP are more likely to meet more health metrics

Our study participants were all free of dyslipidemia, hypertension, and diabetes, which belong to Life's Simple 7 (LS7) (34, 35). Those who also have the optimal PP may meet more health metrics. Those with the optimal PP between 1 SD had a higher proportion of individuals meeting the objectively measured health metrics, such as BMI <25 kg/m^2^ and BP <120/80 mmHg, rather than the subjective items on the questionnaire, compared to groups with PP between ±1~2SD and out of ±2SD. As an objectively measured value, PP based on the difference between SBP and DBP is relatively easy to monitor, compared to some other health metrics. For instance, the LS7 was found to be associated with a 10% lower risk of major adverse cardiovascular events in the Heart Strategies Concentrating on Risk Evaluation (Heart SCORE) study for a community-based sample of adults (36). Establishing personalized optimal PP values for individuals of different ages and sexes will increase the clinical utility and accessibility of PP, and here we tried to demonstrate the application value of mean PP between ±1 SD in a population with favorable CVH.

### People meeting more health metrics are more likely to have the optimal PP

Individuals who meet the BMI and BP standards in our study are more likely to have the optimal PP (Table 4). This is in line with the findings in the Bogalusa Heart Study, in which the association of increased childhood BMI and its cumulative burden with adult arterial stiffness measured as aortic-femoral pulse wave velocity (afPWV) is predominantly mediated through increased BP (37). A 1-point increase in LS7 score was associated with an 8% lower risk of stroke (hazard ratios, 0.92; 95% confidence interval, 0.88–0.95) in the REGARDS study of individuals aged ≥45 years (38). It was concluded that better CVH, based on the LS7 score, is associated with a lower risk of stroke. We have further demonstrated that individuals with a higher Hscore, meeting more items among the five metrics, are more likely to have the optimal PP (Table 4). This could be confirmed by objectively measured BMI and BP for both sexes, but not necessarily by the metrics derived from the subjective questionnaire. BMI and BP, compared to the other three questionnaire-based items, have a higher impact and better determine whether participants have the optimal PP.

### Strengths and limitations

The major strengths of our study include the use of data from a prospective community-based cohort within which individuals with favorable CVH were recruited based on a standardized medical examination program. This ongoing large prospective cohort offers detailed serum biomarkers not available in the LS7, including TG, LDL-C, HDL-C, thyroid, renal, and liver function assessments. The Hscore including the sum of scores for five-health metrics was, therefore, generated to demonstrate that people with a higher Hscore are more likely to have the optimal PP in such a cohort with favorable CVH.

Our study has several limitations. First, direct measurements of arterial stiffness such as afPWV or carotid-femoral PWV (39–41) were not included. Instead, PP was measured as a proxy for arterial stiffness because of its easy accessibility. Second, the lifestyle questionnaire inquiries about physical activity were based on only recall of exercise taking place in the previous 2 weeks. Ideally, physical activity should be defined as the product of metabolic equivalent value and duration of exercise (34, 42). Third, study individuals are subject to confounding by smoking and reverse causality because of preexisting conditions, which might have led to an underestimate of these effects on the other metric values. Fourth, only 4.43% of all female subjects had undergone menopause because more young healthy subjects without menopause who fit the criteria of CVH were recruited in this study. Fifth, the prognosis of these participants is needed to study the predictive value for CVD risks using the proposed optimal PP values. Sixth, our study individuals are a very healthy Chinese population with a lack of generalizability, and a broader population is required to demonstrate the predictive value using the optimal PP.

### Perspectives

Personalized optimal PP based on age and sex is essential for promoting the application of PP measurement in broader populations. We have shown that PP in female subjects becomes greater than in male subjects after the age of 50 years among subjects with favorable CVH. We further demonstrated the relationships between PP and health metrics. Individuals with the optimal PP meet more healthy metrics such as BMI and BP, and vice versa, i.e., individuals meeting more healthy metrics with a higher Hscore are more likely to have the optimal PP. Among common health metrics for CVH, PP is easily accessible and has a greater predictive value for cardiovascular events. But its clinical value, using the optimal values we propose here, remains to be demonstrated in a general population. Longitudinal studies on CVD in our cohort with favorable CVH will provide evidence for the predictive value of the optimal PP and the Hscore proposed in the present study.

## Data availability statement

The original contributions presented in the study are included in the article/Supplementary material, further inquiries can be directed to the corresponding author/s.

## Ethics statement

The study protocols (2021-04-015BC) were approved by the Institutional Review Board of the Taipei Veterans General Hospital (Taipei, Taiwan). The patients/participants provided their written informed consent to participate in this study.

## Author contributions

C-HC, J-HY, T-WC, YC, CL, and Y-FS contributed substantially to the research concept and design, and acquisition, analysis, and interpretation of data. C-HC, Y-KL, F-CY, G-SP, and Y-FS drafted and revised the article to be published. All authors contributed to the article and approved the submitted version.

## Funding

This study was supported by grants from the Ministry of Science and Technology Taiwan (MOST-109-2314-B-016-008), the Ministry of National Defense Medical Affairs Bureau (MND-MAB-C-111-07-111028, MND-MAB-110-028), Tri-Service General Hospital (TSGH-E-111228, TSGH-E-110196, TSGH-E-109226, and TSGH-C108-006-007-007-S05), and Taipei Veterans General Hospital, Hsinchu Branch (2022 VHCT-RD01 and VHCTRD05). Data for this research were sourced from the MJ Health Database.

## Conflict of interest

The authors declare that the research was conducted in the absence of any commercial or financial relationships that could be construed as a potential conflict of interest.

## Publisher's note

All claims expressed in this article are solely those of the authors and do not necessarily represent those of their affiliated organizations, or those of the publisher, the editors and the reviewers. Any product that may be evaluated in this article, or claim that may be made by its manufacturer, is not guaranteed or endorsed by the publisher.
