# Supplementary material for: The optimal pulse pressures for healthy adults with different ages and sexes correlate with cardiovascular health metrics
Source: Front Cardiovasc Med. 2022 Dec 5;9:930443. doi: 10.3389/fcvm.2022.930443 (PMC9760735; doi:10.3389/fcvm.2022.930443)
Supplement: Supplementary file 1 [file Data_Sheet_1.doc]

**Supplementary Table S1. Association Between Pulse Pressure (PP) and the Clinical and Laboratory Characteristics**

| **Male** | **PP, mmHg** | | | | | | | | | | | | | | ***r*** |
| --- | --- | --- | --- | --- | --- | --- | --- | --- | --- | --- | --- | --- | --- | --- | --- |
|  | **<=19** | **20-24** | **25-29** | **30-34** | **35-39** | **40-44** | **45-49** | **50-54** | **55-59** | **60-64** | **65-69** | **70-74** | **75-79** | **>=80** |  |
| SBP, mmHg | 94.1 | 96.9 | 100.2 | 104.0 | 107.9 | 111.6 | 115.9 | 120.6 | 124.9 | 128.7 | 131.1 | 133.3 | 134.6 | 135.8 | 0.6916* |
| DBP, mmHg | 76.9 | 74.4 | 72.9 | 71.8 | 70.7 | 69.5 | 68.9 | 68.8 | 68.2 | 67.1 | 64.5 | 61.8 | 58.3 | 54.2 | -0.1925* |
| BMI, kg/m^2^ | 21.9 | 21.8 | 21.9 | 22.1 | 22.3 | 22.3 | 22.4 | 22.5 | 22.6 | 22.8 | 22.8 | 22.9 | 21.5 | 22.3 | 0.0728* |
| WC, cm | 76.1 | 76.1 | 76.4 | 76.8 | 77.1 | 77.1 | 77.3 | 77.4 | 77.5 | 77.6 | 77.3 | 77.5 | 73.8 | 77.0 | 0.0325* |
| FPG, mg/dL | 94.6 | 94.6 | 95.0 | 95.4 | 95.8 | 96.0 | 96.1 | 96.2 | 96.4 | 96.0 | 95.6 | 95.7 | 96.8 | 93.1 | 0.0389* |
| AST, I/L | 20.1 | 20.0 | 19.6 | 19.7 | 19.6 | 19.4 | 19.4 | 19.3 | 19.1 | 19.1 | 19.0 | 19.1 | 18.2 | 20.0 | -0.0547* |
| ALT, U/L | 20.2 | 19.9 | 19.5 | 19.6 | 19.6 | 19.6 | 19.6 | 19.6 | 19.3 | 19.3 | 19.0 | 18.7 | 17.5 | 18.8 | -0.0170* |
| GGT, U/L | 17.0 | 18.2 | 18.0 | 18.0 | 18.2 | 18.1 | 18.1 | 18.0 | 17.8 | 17.4 | 17.7 | 17.2 | 16.4 | 15.7 | -0.0155* |
| TG, mg/dL | 80.7 | 81.7 | 80.3 | 81.3 | 81.7 | 80.2 | 79.8 | 79.1 | 79.0 | 77.3 | 76.6 | 74.5 | 76.2 | 68.2 | -0.0390* |
| CHOL, mg/dL | 172.1 | 170.9 | 169.7 | 169.7 | 170.0 | 169.9 | 169.5 | 168.5 | 168.4 | 167.8 | 165.6 | 165.3 | 166.1 | 157.1 | -0.0410* |
| HDL-C, mg/dL | 54.8 | 56.1 | 55.6 | 55.3 | 54.9 | 54.7 | 54.6 | 54.5 | 54.2 | 54.2 | 54.3 | 54.7 | 56.0 | 52.5 | -0.0326* |
| LDL-C, mg/dL | 101.6 | 98.4 | 98.5 | 98.5 | 99.0 | 99.3 | 99.1 | 98.4 | 98.3 | 98.1 | 95.9 | 95.8 | 94.8 | 91.5 | -0.0175* |
| TSH, pg/dL | 1.4 | 1.4 | 1.5 | 1.4 | 1.4 | 1.4 | 1.4 | 1.4 | 1.5 | 1.4 | 1.5 | 1.4 | 1.4 | 1.8 | 0.0031 |
| eGFR, mL/min/1.73m^2^ | 83.2 | 84.2 | 83.9 | 84.3 | 84.9 | 85.7 | 86.4 | 87.4 | 88.1 | 88.9 | 90.0 | 88.8 | 91.1 | 91.0 | 0.1106* |

| **Female** | **PP, mmHg** | | | | | | | | | | | | | | ***r*** |
| --- | --- | --- | --- | --- | --- | --- | --- | --- | --- | --- | --- | --- | --- | --- | --- |
|  | **<=19** | **20-24** | **25-29** | **30-34** | **35-39** | **40-44** | **45-49** | **50-54** | **55-59** | **60-64** | **65-69** | **70-74** | **75-79** | **>=80** |  |
| SBP, mmHg | 90.3 | 92.9 | 95.6 | 98.6 | 101.8 | 105.2 | 110.2 | 115.7 | 121.3 | 125.8 | 128.8 | 130.8 | 132.4 | 135.3 | 0.6885* |
| DBP, mmHg | 73.4 | 70.5 | 68.3 | 66.6 | 64.7 | 63.3 | 63.3 | 63.9 | 64.6 | 64.3 | 62.2 | 59.3 | 55.9 | 52.8 | -0.1696* |
| BMI, kg/m^2^ | 20.4 | 20.4 | 20.5 | 20.7 | 20.8 | 20.8 | 21.1 | 21.5 | 21.7 | 21.8 | 22.0 | 21.6 | 21.7 | 22.6 | 0.1261* |
| WC, cm | 67.3 | 67.0 | 67.4 | 67.7 | 67.9 | 68.1 | 68.6 | 69.3 | 69.7 | 70.1 | 70.4 | 69.5 | 69.6 | 71.8 | 0.1133* |
| FPG, mg/dL | 91.0 | 91.3 | 91.4 | 91.6 | 91.7 | 92.2 | 92.6 | 93.1 | 93.5 | 93.6 | 93.5 | 92.8 | 93.4 | 94.5 | 0.0914* |
| AST, I/L | 17.8 | 17.7 | 17.9 | 17.6 | 17.6 | 17.6 | 17.6 | 17.7 | 17.8 | 17.8 | 18.0 | 17.9 | 18.7 | 17.3 | 0.0041 |
| ALT, U/L | 14.2 | 14.2 | 14.5 | 14.4 | 14.4 | 14.5 | 14.6 | 14.9 | 15.1 | 15.0 | 15.3 | 14.8 | 15.7 | 13.5 | 0.0396* |
| GGT, U/L | 12.4 | 12.1 | 12.4 | 12.4 | 12.4 | 12.4 | 12.5 | 12.8 | 13.0 | 12.6 | 13.0 | 12.6 | 12.0 | 12.5 | 0.0270* |
| TG, mg/dL | 67.4 | 66.4 | 66.9 | 67.1 | 66.7 | 67.2 | 67.8 | 69.3 | 70.8 | 70.7 | 70.8 | 70.4 | 79.2 | 70.6 | 0.0429* |
| CHOL, mg/dL | 167.4 | 169.4 | 168.8 | 168.4 | 168.8 | 169.0 | 169.2 | 170.1 | 170.9 | 169.6 | 171.4 | 170.7 | 171.7 | 167 | 0.0280* |
| HDL-C, mg/dL | 62.3 | 63.0 | 62.3 | 61.9 | 62.0 | 62.0 | 61.7 | 61.4 | 61.2 | 60.7 | 60.5 | 62.3 | 59.7 | 60.1 | -0.0281* |
| LDL-C, mg/dL | 91.8 | 93.2 | 93.1 | 93.1 | 93.5 | 93.7 | 93.9 | 94.8 | 95.4 | 94.7 | 96.7 | 94.1 | 96.2 | 93.4 | 0.0355* |
| TSH, pg/dL | 1.5 | 1.6 | 1.6 | 1.5 | 1.5 | 1.5 | 1.5 | 1.6 | 1.6 | 1.6 | 1.5 | 1.6 | 1.6 | 1.6 | 0.0073* |
| eGFR, mL/min/1.73m^2^ | 88.6 | 88.6 | 88.2 | 89.0 | 89.5 | 91.3 | 90.5 | 90.4 | 90.2 | 90.7 | 90.1 | 89.6 | 91.0 | 86.8 | 0.0100* |

BMI indicates body mass index; WC, waist circumference; SBP, systolic blood pressure; DBP, diastolic blood pressure; PP, pulse pressure; FPG, fasting plasma glucose; AST, aspartate aminotransferase; ALT, alanine aminotransferase; GGT, gamma glutamyl transferase; eGFR, estimated glomerular filtration rate; TG, triglyceride; CHOL, total cholesterol, HDL-C, high-density lipoprotein cholesterol; LDL-C, low-density lipoprotein cholesterol; TSH, thyroid-stimulating hormone. *p<0.05; **p<0.01; ***p<0.001

**Supplementary Table S2.** Association Between Pulse Pressure (PP) and Health Metrics

(A)

|  | **Male** | | | | **Female** | | | |
| --- | --- | --- | --- | --- | --- | --- | --- | --- |
|  | **PP between ±1 SD** | **PP between ±1~2 SD** | **PP**  **out of**  **±2 SD** | **Total** | **PP between ±1 SD** | **PP between ±1~2 SD** | **PP**  **out of**  **±2 SD** | **Total** |
| **Smoking** | χ^2^=0.064 | | | | χ^2^=48.76*** | | | |
| **Yes** | 8,685 | 3,318 | 575 | 12,578 | 5,506 | 2,026 | 319 | 7,851 |
|  | 30.01% | 29.88% | 29.98% | 29.98% | 7.14% | 6.04% | 6.05% | 6.77% |
| **No** | 20,252 | 7,785 | 1,343 | 29,380 | 71,638 | 31,493 | 4,953 | 108,084 |
|  | 69.99% | 70.12% | 70.02% | 70.02% | 92.86% | 93.96% | 93.95% | 93.23% |
| **Total** | 28,937 | 11,103 | 1,918 | 41,958 | 77,144 | 33,519 | 5,272 | 115,935 |
|  | 100.00% | 100.00% | 100.00% | 100.00% | 100.00% | 100.00% | 100.00% | 100.00% |
|  |  |  |  |  |  |  |  |  |
| **Physical activity** | | χ^2^=1.81 | | | χ^2^=101.99*** | | | |
| **No** | 10,385 | 3,901 | 685 | 14,971 | 39,677 | 16,074 | 2,692 | 58,443 |
|  | 36.68% | 35.97% | 36.87% | 36.50% | 52.30% | 48.99% | 52.17% | 51.34% |
| **Yes** | 17,926 | 6,943 | 1,173 | 26,042 | 36,189 | 16,738 | 2,468 | 55,395 |
|  | 63.32% | 64.03% | 63.13% | 63.50% | 47.70% | 51.01% | 47.83% | 48.66% |
| **Total** | 28,311 | 10,844 | 1,858 | 41,013 | 75,866 | 32,812 | 5,160 | 113,838 |
|  | 100.00% | 100.00% | 100.00% | 100.00% | 100.00% | 100.00% | 100.00% | 100.00% |
|  |  |  |  |  |  |  |  |  |
| **Heathy diet score** | | χ^2^=2.52 | | | χ^2^=121.80*** | | | |
| **<2** | 15,254 | 5,811 | 1,047 | 22,112 | 33,532 | 13,215 | 2,066 | 48,813 |
|  | 52.24% | 51.91% | 53.86% | 52.22% | 45.13% | 41.55% | 42.25% | 43.98% |
| **≥2** | 13,948 | 5,383 | 897 | 20,228 | 40,773 | 18,589 | 2,824 | 62,186 |
|  | 47.76% | 48.09% | 46.14% | 47.78% | 54.87% | 58.45% | 57.75% | 56.02% |
| **Total** | 29,202 | 11,194 | 1,944 | 42,340 | 74,305 | 31,804 | 4,890 | 110,999 |
|  | 100.00% | 100.00% | 100.00% | 100.00% | 100.00% | 100.00% | 100.00% | 100.00% |

(B)

|  | **Male** | | | | **Female** | | | |
| --- | --- | --- | --- | --- | --- | --- | --- | --- |
|  | **PP between ±1 SD** | **PP between ±1~2 SD** | **PP**  **out of**  **±2 SD** | **Total** | **PP between ±1 SD** | **PP between ±1~2 SD** | **PP**  **out of**  **±2 SD** | **Total** |
| **Smoking** | χ^2^=0.064 | | | | χ^2^=48.76*** | | | |
| **Yes** | 8,685 | 3,318 | 575 | 12,578 | 5,506 | 2,026 | 319 | 7,851 |
|  | 69.05% | 26.38% | 4.57% | 100% | 70.13% | 25.81% | 4.06% | 100% |
| **No** | 20,252 | 7,785 | 1,343 | 29,380 | 71,638 | 31,493 | 4,953 | 108,084 |
|  | 68.93% | 26.50% | 4.57% | 100% | 66.28% | 29.14% | 4.58% | 100% |
| **Total** | 28,937 | 11,103 | 1,918 | 41,958 | 77,144 | 33,519 | 5,272 | 115,935 |
|  |  |  |  |  |  |  |  |  |
| **Physical activity** | | χ^2^=1.81 | | | χ^2^=101.99*** | | | |
| **No** | 10,385 | 3,901 | 685 | 14,971 | 39,677 | 16,074 | 2,692 | 58,443 |
|  | 69.37% | 26.06% | 4.58% | 100% | 67.89% | 27.50% | 4.61% | 100% |
| **Yes** | 17,926 | 6,943 | 1,173 | 26,042 | 36,189 | 16,738 | 2,468 | 55,395 |
|  | 68.83% | 26.66% | 4.50% | 100% | 65.33% | 30.22% | 4.46% | 100% |
| **Total** | 28,311 | 10,844 | 1,858 | 41,013 | 75,866 | 32,812 | 5,160 | 113,838 |
|  |  |  |  |  |  |  |  |  |
| **Heathy diet score** | | χ^2^=2.52 | | | χ^2^=121.80*** | | | |
| **<2** | 15,254 | 5,811 | 1,047 | 22,112 | 33,532 | 13,215 | 2,066 | 48,813 |
|  | 68.99% | 26.28% | 4.73% | 100.00% | 68.69% | 27.07% | 4.23% | 100.00% |
| **≥2** | 13,948 | 5,383 | 897 | 20,228 | 40,773 | 18,589 | 2,824 | 62,186 |
|  | 68.95% | 26.61% | 4.43% | 100.00% | 65.57% | 29.89% | 4.54% | 100.00% |
| **Total** | 29,202 | 11,194 | 1,944 | 42,340 | 74,305 | 31,804 | 4,890 | 110,999 |

SD indicates standard deviation. *p<0.05; **p<0.01; ***p<0.001

**Supplementary Table S3.** Multiple stepwise regression analysis for variables of pulse pressure

| Variable | Pulse pressure | | |
| --- | --- | --- | --- |
|  | Model1 | Model2 | Model3 |
| Sex | -3.764*** | -3.764*** | -3.152*** |
|  | (0.053) | (0.053) | (0.054) |
| Age |  | -0.008** | -0.037*** |
|  |  | (0.003) | (0.003) |
| BMI |  |  | 0.417*** |
|  |  |  | (0.009) |
| Constant | 49.359*** | 49.635*** | 40.677*** |
|  | (0.095) | (0.133) | (0.230) |
| N | 162,636 | 162,636 | 162,636 |
| R-sq | 0.030 | 0.030 | 0.043 |
| Adj. R-sq | 0.030 | 0.030 | 0.043 |

BMI indicates body mass index; Standard errors are in parentheses. * p<0.05, ** p<0.01, *** p<0.001.


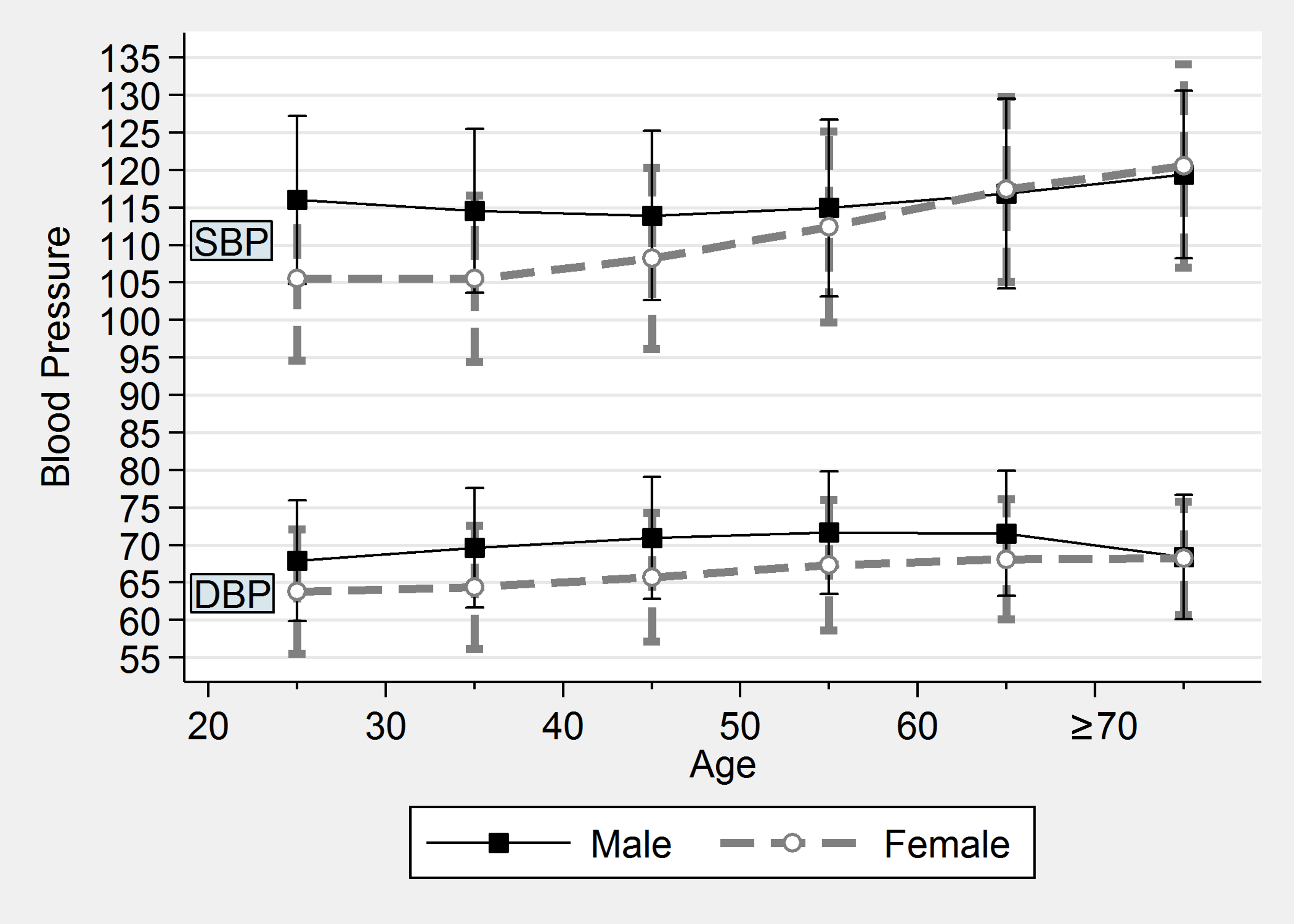


**Supplementary Figure S1. Blood pressure by sex and age.** Systolic blood pressure (SBP) increases across the age range in females, in contrast with relatively stable SBP in males. Diastolic blood pressure (DBP) increases until 50 years of age and thereafter declines in males but not in females.
